# Hyperthermia combined with immune checkpoint inhibitor therapy in the treatment of primary and metastatic tumors

**DOI:** 10.3389/fimmu.2022.969447

**Published:** 2022-08-12

**Authors:** Ximing Yang, Miaozhi Gao, Runshi Xu, Yangyang Tao, Wang Luo, Binya Wang, Wenliang Zhong, Lan He, Yingchun He

**Affiliations:** ^1^ Medical School, Hunan University of Chinese Medicine, Changsha, China; ^2^ Hunan Provincial Ophthalmology and Otolaryngology Diseases Prevention and Treatment with Traditional Chinese Medicine and Visual Function Protection Engineering and Technological Research Center, Changsha, China; ^3^ Hunan Provincial Key Laboratory for the Prevention and Treatment of Ophthalmology and Otolaryngology Diseases with Traditional Chinese Medicine, Changsha, China; ^4^ The First Hospital of Hunan University of Chinese Medicine, Changsha, China

**Keywords:** thermal ablation, mild hyperthermia, immune checkpoint inhibitor, malignant tumor, combined therapy

## Abstract

According to the difference in temperature, thermotherapy can be divided into thermal ablation and mild hyperthermia. The main advantage of thermal ablation is that it can efficiently target tumors in situ, while mild hyperthermia has a good inhibitory effect on distant metastasis. There are some similarities and differences between the two therapies with respect to inducing anti-tumor immune responses, but neither of them results in sustained systemic immunity. Malignant tumors (such as breast cancer, pancreatic cancer, nasopharyngeal carcinoma, and brain cancer) are recurrent, highly metastatic, and highly invasive even after treatment, hence a single therapy rarely resolves the clinical issues. A more effective and comprehensive treatment strategy using a combination of hyperthermia and immune checkpoint inhibitor (ICI) therapies has gained attention. This paper summarizes the relevant preclinical and clinical studies on hyperthermia combined with ICI therapies and compares the efficacy of two types of hyperthermia combined with ICIs, in order to provide a better treatment for the recurrence and metastasis of clinically malignant tumors.

## Introduction

Hyperthermia (HT) appears more and more in the clinical comprehensive treatment strategy of tumor. There are many clinical benefits of hyperthermia, especially its ability to regulate immune responses. Immunotherapy has shed light on a new form of tumor therapy. New immune checkpoint inhibitors (ICIs) have been developed as treatment strategies to target the immune evasion characteristics of tumors (1). Immunotherapy, especially PD-1 and anti-PD-L1 antagonists, has shown therapeutic effects on many malignant tumors. However, the response rate of immunotherapy is still not high, and the most lasting immune response is only observed in a small number of patients. The activation and maintenance of anti-tumor immune response depends on immune cells, such as dendritic cell (DC), natural killer cell (NK), T lymphocytes, B lymphocytes. First, tumor-associated antigens are recognized by DCs, with the latter becoming mature and activated; then, mature DCs present antigens to the initial T cells, which are activated into effector T cells, especially cytotoxic CD8+T cells, infiltrating distant tumors and kill the remaining tumor cells (2–4). NK cells are activated in a manner similar to that of CD8+T cells by releasing cytotoxic particles containing perforin and granzyme to directly lyse tumor cells. At the same time, NK cells are important in regulating adaptive immune response because of their ability to release interferon-γ (IFN–γ) and tumor necrosis factor-α (TNF–α) (5–7). In addition, some studies have shown that the expression of B cell-related genes, such as MZB1, JCHAIN and IGLL5, is significantly increased in patients who respond to ICI therapy, suggesting the potential of B cells in improving anti-tumor immune response (8). However, Tumor cells evade immunity through the expression of programmed cell death ligand (pdl-1) and similar inhibitory gene products, such as indoleamine 2 and 3 dioxygenase (Ido), transforming growth factor-β (TGF-β) and interleukin-10 (IL-10) (9). One of the important mechanisms of tumor immune escape is the inactivation or silencing of effector T cells. the depletion of T cells is closely related to inhibitory receptors, such as programmedcelldeathprotein-1 (PD-1), TIM-3 (mucin3) and LAG-3 (Lymphocyteactivationgeneprotein-3) (10, 11). Therefore, these receptors and their corresponding ligands form new immune checkpoints. At present, the treatment strategy of ICIs is mainly targeted at T cells, but the response rate of patients to ICIs is often not high. Based on the low response rate of immunotherapy, combination therapy has been proposed to provide a lasting systemic anti-tumor immune response. Among these combined strategies, it is surprising to find that the combination of HT and ICIs may enhance the anti-tumor immune response (2). Therefore, at present, the research on the combination of them in the treatment of malignant tumors is a hot spot.

In 1927, Wagner-Jauregg won the Nobel Prize in Physiology and Medicine for the use of hyperthermia. He found that the high fever associated with malaria hindered the infectious factor and, through this study, he was able to identify the preliminary role of hyperthermia. Previously, hyperthermia was defined as a core body temperature greater than 41 °C for more than 60 minutes (12). Currently, according to the temperature, hyperthermia therapy can be further divided into two sub treatments: thermal ablation and mild hyperthermia (13).

Thermal ablation causes direct necrosis in the central area of the tumor by increasing the temperature in the tumor to more than 55 °C. However, the temperature during mild hyperthermia is relatively low, and tumor cell damage is often induced in the range of 41 to 45 °C (14). The temperature difference between the two types of hyperthermia and their different effects often lead to different therapeutic outcomes. At the same time, thermal ablation is similar to surgical treatment, mostly disposable treatment; mild hyperthermia, due to the low energy input, usually requires repeated, longer heating time (> 30 min) to achieve any effect (15). Mild hyperthermia presents a more tedious regimen, and the killing effect on tumor needs to be studied. However, like thermal ablation, mild hyperthermia also can elicit immune responses, and several studies demonstrate that the immune responses to thermal ablation may have been induced by mild hyperthermia. The connection between the immune responses to thermal ablation and mild hyperthermia have garnered interest and further investigation is warranted. Current literature lacks studies on the intersection and comparison of the two therapies in anti-tumor treatment; hence, this paper provides a reference for the better use of the combination of hyperthermia and ICI therapy by comparing the different immune responses brought by various hyperthermia methods.

## Effects of thermal ablation and mild hyperthermia on the immune response

Both thermal ablation and mild hyperthermia use heat to destroy tumor tissue, but the effects of the two treatments on the immune response may vary. After thermal ablation, the tumor tissue can be divided into the central area (> 55 °C), transitional zone (41 °C-45 °C), and normal tissue area (16). Coagulative necrosis often occurs in the central area, leading to the release of new antigens in tumor cells, which is called tumor *in situ* vaccination, while the transition zone is easily affected by residual temperature, resulting in secondary effects such as immune activation (17). However, the latest research shows that thermal ablation not only irreversibly denatures the newly released antigens in the central area of tumor tissues but may also upregulate some carcinogenic factors (such as interleukin-6(IL-6) and vascular endothelial growth factor(VEGF)), aggravating the progression of tumor metastasis (18, 19). However, thermal ablation still has some positive immunomodulatory effects, such as the release of positive immunomodulatory factors, initiation of a local inflammatory response, and recruitment of immune cells (18, 20). Compared with thermal ablation, the main effect of mild hyperthermia is not necrosis, but apoptosis, vascular permeability, and immune effect. Among them, the effect of mild hyperthermia on tumor immune response is mainly seen *via* an increase in immunogenicity, promotion of innate immune cell infiltration, and improvement in the tumor microenvironment (TME) (21–23).

The above processes involve all aspects of an immune response. Thermal ablation and mild hyperthermia can respectively induce a series of immune-related factors to enhance the immunogenicity and immunoreactivity of ICIs in the TME (24–26). Next, we will explore the complementary effects of hyperthermia on ICIs from several angles, namely, the effects on immunogenicity, on immune cell activation, and on the immunosuppressive TME.

## Effect on immunogenicity

Immunogenicity is the basis of an anti-tumor immune response, which requires two aspects in order to be initiated: the existence of antigen library and the occurrence of an immunogenic reaction (27). According to the consensus guidelines for immunogenic cell death (ICD), the presence of antigens alone is not sufficient to initiate an anti-tumor immune response, which requires the presence of ICD (28).

On the one hand, in many preclinical studies, thermal ablation has been proved to have the ability to release tumor associated antigens (TAAs). Ghanamah et al. and Leibovici et al. observed transient increases in carcinoembryonic antigen (CEA) and prostate specific antigen (PSA) levels in colorectal cancer and pancreatic cancer, respectively (29, 30). Among them, 59% of colorectal cancer patients (10/17) had a rapid increase in CEA levels 1 day after thermal ablation, suggesting that thermal ablation accelerated the tumor immune cycle. However, too high of a temperature during thermal ablation often leads to degeneration of new tumor antigens. Mild hyperthermia has an advantage in this respect, because a temperature range of 41 to 45 °C is insufficient to cause antigenic degeneration. Ruoping et al. reported a method of inducing mild hyperthermia based on copper sulfide nanoparticles (CuSNPs), which enhanced the capture of tumor antigens released during hyperthermia and induced the tumor immunogenic microenvironment, suggesting the potential of mild hyperthermia in improving immunogenicity (31).

On the other hand, thermal ablation mainly caused cell necrosis, while mild hyperthermia mainly caused apoptosis (32, 33). The advantage of inducing apoptosis over direct necrosis is that more damage-related molecules (DAMPs) are released during apoptosis to induce ICD (34). Studies have shown that mild hyperthermia induces ICD in a ROS-dependent manner, which is evidenced by the occurrence of a key ICD event: phosphorylation of eIF2 α (35, 36). Currently, there is no direct evidence of ICD induction by thermal ablation, but the effects on immune cell activation are clear.

## Effect on the activation and action of immune cells

Previously, it was believed that the efficacy of thermal ablation primarily depended on its ability to ablate the tumor *in situ* (37). In fact, thermal ablation has been increasingly used in the treatment of metastatic tumors depending on its secondary effects, such as activating anti-tumor immune responses. Mature dendritic cells (DCs) play a key role before T cells are recruited, recognize tumor cells, and infiltrate the tumor site. After thermoablation, DC and natural killer (NK) cells are recruited to the tumor site, while regulatory T cells (Tregs) are significantly downregulated (38). Muxin et al. confirmed that hyperthermia inhibits the progression of primary and pulmonary metastasis of breast cancer by activating the macrophage/IL-15/NK cell axis (39). However, Muxin et al. and other researchers have not observed significant T cell immune response (40, 41), suggesting that Tregs may play a key role in the efficacy of thermal ablation, but the role of other T cells in it is still worthy of attention.

It is well known that mild hyperthermia can improve vascular permeability, which provides a good platform for the passage of innate immune cells and lays a foundation for inhibiting distant metastasis of tumors (42, 43). Yuefei et al. found that mild hyperthermia not only activated macrophages and DCs in tumors but also improved the structure of tumor vessels by reducing the levels of transforming growth factor-β (TGF-β) and hypoxia-inducible factor-1 α (HIF-1α) and significantly increased the proportion of CD8+IFN- γ + T cells (44). In breast cancer, Wan et al. found a significant increase and infiltration of DC, NK, B and CD8+T cells after mild hyperthermia (45), while Muxin et al. did not observe a significant T cell response induced by hyperthermia (39). Thus, at least in breast cancer, mild hyperthermia may lead to more effective immune stimulation and longer-lasting immune memory than thermal ablation.

## Influence on the immunosuppressive TME

The efficacy of ICIs in the treatment of malignant tumors depends, to a large extent, on the immune status of the TME (46, 47). According to the immune status of the TME, tumors can be divided into “cold” and “hot” tumors. The characteristics of “cold” tumors include less tumor infiltrating T cells (TIL), low expression of PD-L1, enrichment of immunosuppressive cells, and decrease in tumor mutation, so the response rate to PD-1/PD-L1mAb is usually low (48). In other words, if thermal ablation or mild hyperthermia changed some of the characteristics of “cold” tumors, it could increase the sensitivity of ICIs, which would help improve the efficacy of this therapy in conjunction with ICIs.

The infiltration of TILs and the reduction of immunosuppressive cells (such as Tregs) are often considered to be a temperature-sensitive event. Liping et al. designed a mild hyperthermia method combined with PD-L1 inhibitors for the “cold” TME of breast cancer mouse models (4T1 and B16F10). The results showed that the CD8+T and CD4+T cell populations in the treatment group were 11.8 and 8.2 times higher than those in the PBS group, respectively, promoting the differentiation of immature T cells (49). More importantly, the infiltration of CD8+T cells was also significantly increased, and the number of Tregs and the level of Treg surface markers such as CD4, CD25, and Foxp3 were significantly decreased. Interestingly, they found that the expression of PD-L1 on the surface of tumor cells increased with the increase in temperature in the range of 37–45°C, suggesting the sensitizing effect of mild hyperthermia on ICIs. In addition to these, Changdong et al. also found that whole body hyperthermia (WBH) has a good effect on α4β1 or α4β7 integrin-mediated T cell adhesion and migration (50), which may be one of the mechanisms by which hyperthermia inhibits distant tumor metastasis.

## Magnetic nanoparticles as thermal effectors and drug carriers

Modified nanoparticles play a key role in the combined treatment strategy of cancer based on magnetic hyperthermia(MHT) and immunotherapy. Superparamagnetic iron oxide nanoparticles (SPIONs) smaller than 20-25 nm have attracted much attention because of their good superparamagnetism, chemical stability, high saturation magnetization and proper biocompatibility (51). Based on the above characteristics, the coupling of SPION with drugs can better maintain the retention time of drugs in the blood, control the degradation rate of drugs and reduce toxicity, suggesting that this coupling is helpful for better targeted delivery of drugs to tumor sites, and provides the possibility for the combination of magnetic hyperthermia and immunotherapy (52). However, it is worth noting that this targeted transport often depends on monoclonal antibodies to recognize the receptors overexpressed on the cell surface, in which SPION mainly plays the role of a carrier, in other words, it is difficult to target nanoparticles at low-specific tumor locations.

The combination of magnetic nanoparticles (MNPs) and alternating magnetic field (AMF) is called MHT, which is essentially a kind of thermotherapy with magnetic induction characteristics (53). SPIONs belongs to a kind of MNP, Tetsuya. After coupling SPION with anti-HER2 antibody trastuzumab, it was found that anti-HER2SPIONS selectively targeted HER2-expressing cancer cells and induced apoptosis only in cancer cells expressing HER2, suggesting the specificity of this coupling against cancer (53). Not only that, SPION also performs well in high intensity focused ultrasound (HIFU). Compared with HIFU therapy alone, Haiyan et al. invented a kind of nanoparticles with superparamagnetic iron oxide (SPIO,Fe3O4NPs) as shell and poly (lactide-glycolide) nanoparticles (Fe3O4@PLGA/LANPs) as core, which showed synergistic inhibitory effect on breast cancer, which was related to the accumulation and long-term retention of SPION in tumor site (54). Many MNP are evolved on the basis of SPION, other MNP including CoFe2O4@MnFe2O4, PEGylatedFeNP, etc., can also provide magnetic targeting efficacy, but compared with SPION, some nanoparticles that are too large or too small cannot avoid liver uptake (55).

However, some questions about the efficacy of hyperthermia are also emerging. For example, tumor cells often produce heat resistance during or after hyperthermia, which is characterized by resistance and regenerative response to high temperature (51). The reason behind this phenomenon is closely related to the enhancement of heat shock protein synthesis (56). Although HSP70 and many other HSPs have immunostimulatory effects, there is considerable evidence that some HSPs inhibit the anti-tumor immune response (57, 58). The targets of SPIONs include hsp70 and hsp90, which may help to prevent the production of heat tolerance and restore the anti-tumor immune response.

## Role of exosomes as mediators in the hyperthermia-induced immune response

Exosome, a small membrane vesicle derived from endocytosis, mediates cellular communication between tumor cells and immune cells by wrapping and transmitting signal molecules such as mRNA, miRNA and other non-coding RNA and proteins. Among them, HSP has attracted much attention because of its diverse mechanisms of expression and regulation of tumorigenesis and development. During hyperthermia, cells under heat stress enhance immune response by promoting the release of more exosomes rich in tumor antigens, chemokines (including CCL2, CCL3, CCL4, CCL5 and CCL20, etc.) and HSP to APC to identify and destroy tumor cells (59). The contents of TEX (HS-TEX) under heat stress play an important role in its function in TME. Compared with apoptotic fragments and HSP-70 knockout exosomes, HSP-70-rich exosomes recruit more NK cells and promote the killing of NK cells (60). In addition, recent studies have found that exosomes extracted from heat-stressed tumor cells (HS-TEX) can reverse immunosuppressive TME from the following four aspects, including: ① activation of DCs;② as a cancer vaccine promotes IL-6;③ secretion by cells to reduce the proportion of Treg and MDSCs, while increasing the proportion of Th1 and Th17;④ promote the differentiation of CD8+T cells (60–62).

However, exosomes can also produce immunosuppressive activity. Some TEX have been shown to contain ligands such as FasL and TRAIL, which can induce apoptosis of activated T cells. Exosomes can also contain NKG2D ligands, which can block NKG2D receptors and inhibit the cytotoxicity of NK cells and CD8+T cells. One study also showed that HS-TEX can induce a bystander effect (BE) in tumor cells and promote the survival of stress-free cells (63). In addition, PD-L1 can be expressed in TEX to evade immunity (64).

**Figure 1:**
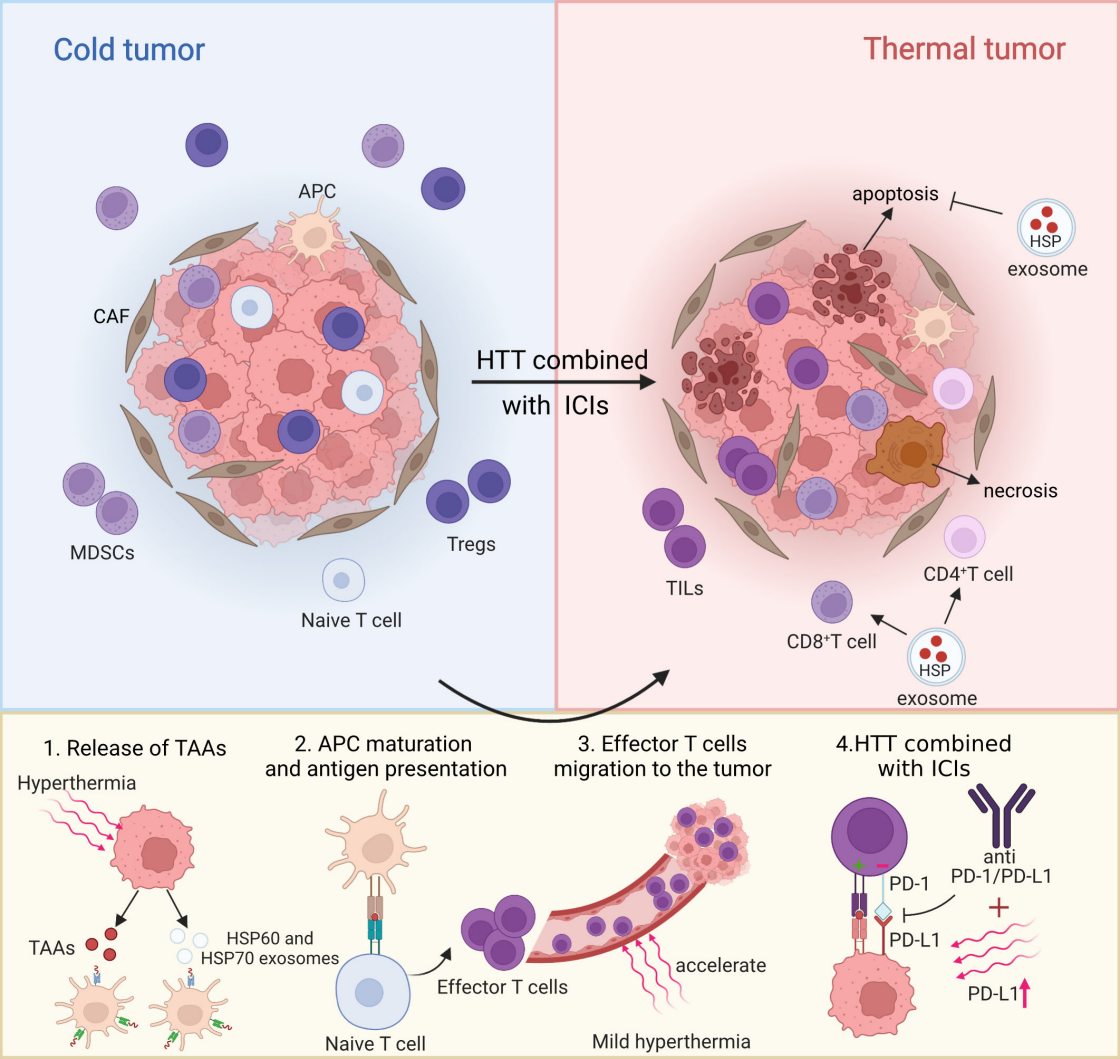
Partial mechanism diagram of hyperthermia combined with ICIs on tumor microenvironment. Created with BioRender.com.

It is worth noting that recently, it has been found that exosomes not only participate in the regulation of immune response, but also promote the survival of thermostable cells. The survival of thermostable cells is inseparable from a mechanism called “Anastasis”, through which cells can recover from apoptotic lesions and have their previous functional state, which is closely related to the role of HSP released by TEX in the fight against apoptosis and lethal stimuli. The increase of Hsp70 and Hsp90, a common feature of thermostable cells, also confirms this point. A recent study also reported that HSP70, HSP90 and HSP60 are secreted by cancer cells through exocytosis and may play a key role in inhibiting the host’s anti-tumor immune response (65). Moreover, this heat tolerance mediated by TEX may be achieved by inhibiting apoptosis by HSP, including inhibition of cascade activation of caspase and splicing of t-BID fragments, which is antagonistic to apoptosis induced by mild hyperthermia. However, TEX rich in HSPs can also induce anti-tumor immunity. For example, studies have suggested that exosome from heat-treated malignant ascites from gastric cancer patients are rich in HSP70 and HSP60, which promote DC maturation and induce tumor-specific CTL response (62). Generally speaking, the key to find a breakthrough between immune stimulation and immunosuppression and the emergence or disappearance of heat tolerance may lie in the role of exosome contents, especially the complex role of HSPs remains to be further studied. In summary, the above data suggest that hyperthermia can be used as an adjuvant therapy for ICIs to temporarily activate the anti-tumor immune response (66). Therefore, several studies propose a therapeutic strategy of TILs infiltration in tumor microenvironment (60), up-regulated expression of PD-L1 and combined with ICIs after thermal ablation or mild hyperthermia, to investigate whether these therapies could elicit stronger and more lasting systemic immunity in tumor patients to resolve recurrence and distant metastasis after hyperthermia (**Figure 1**).

## Thermal ablation or mild hyperthermia combined with ICIs: A preclinical study

The earlier section was only meant to be a general introduction to the immune effects of hyperthermia. Here, we compared the combined effects of each hyperthermia technique in more detail, in order to investigate which one was more efficacious. Thermal ablation mainly included microwave ablation (MWA), radiofrequency ablation (RFA), cryoablation (CA),high intensity focused ultrasound (HIFU) and laser ablation (67, 68). Mild hyperthermia therapies were mainly photothermal therapy (PTT) and magnetothermotherapy (MHT) (69). PTT uses near infrared (NIR) light in the range between 700nm and 900nm to generate thermal energy, including gold, copper, graphene, polymer nanoparticles and carbon nanotubes under study (70–74). This characteristic of near-infrared light determines that PTT can maintain a penetration depth of approximately 1 cm, therefore PTT is more suitable for subcutaneous tumors, surgical exposures, or catheters. MHT uses magnetic nanoparticles (mainly iron oxide (Fe2O3) nanoparticles and its derivatives) to create alternating magnetic fields to generate heat. Compared with PTT, MHT is advantageous because it has excellent tissue penetration for deep tumors using hyperthermia induced by alternating magnetic fields. However, this may also lead to nanoparticles not being located in tumors to unnecessarily heat healthy tissue (75, 76).

Currently, the commonly used immune checkpoint inhibitors approved by FDA included anti-PD-1/PD-L1 inhibitors and anti-CTLA-4 inhibitors (77). Malignant tumors often use the inhibitory PD-1/PD-L1 or CTLA-4 pathway to escape the immune system. The principle of this biological behavior is that the PD-L1 molecule located on the surface of tumor cells binds to the transmembrane protein PD-1 expressed on T cells, B cells and natural killer cells (NK cells), which depletes T cells and promotes immune escape (78). On the other hand, CTLA-4 molecules block the binding of B7 ligands to T cell costimulatory molecules by binding to CD80 and CD86, thus blocking intracellular signal transmission and ultimately specifically preventing T cell activation and proliferation. Therefore, PD-1/PD-L1 blockers and CTLA-4 inhibitors play different roles by blocking parallel but different pathways on tumor cells (9). Common PD-1 antagonists include nivolumab and pembrolizumab, PD-L1 antagonists include atezolizumab and durvalumab, and CTLA-4 antagonists include ipilimumab, temlimumab (79, 80). A single antagonist is not frequently used because of the low patients’ response rate. The combination of two checkpoint inhibitors, such as CTLA and PD-1, has shown some efficacy in melanoma, renal cell carcinoma, non-small cell lung cancer and other malignant tumors. However, treatment of metastatic tumors, which is the real cause of death in most clinical tumor patients, is still not effective, so researchers have shifted their attention from the sequential treatment of single or multiple ICIs to the combination of ICIs and other treatments (11). The combination of ICIs and hyperthermia has performed well in some preclinical studies and may be used as a potential combination therapy to improve the response rate and survival rate in the clinical setting. Next, combined with the characteristics of each tumor, this paper will specifically describe the combined effect of thermal ablation or mild hyperthermia and ICIs, especially in the treatment of metastatic tumors.

## Breast cancer

Triple negative breast cancer (TNBC) is a special subtype of breast cancer, accounting for 15%-20% of all breast cancers. It has strong invasive clinical characteristics and an early high recurrence rate (81). Chemotherapy is the standard treatment for TNBC, but the overall prognosis is poor, with a median overall survival time (OS) of only 12-18 months, while hyperthermia combined with immunotherapy seems to provide better prognosis in the preclinical model of TNBC (82). The tumor formed by 4T1 cells in BALB/c mice is highly similar to the one found in human breast cancer in terms of growth and metastasis, so it is often used as an animal model for the study of human stage IV breast cancer. At the same time, it is also a highly invasive and metastatic model, often with lung, liver, and lymph node metastasis, in line with the characteristics of TNBC (83).

The main advantage of thermal ablation is that it has a strong *in situ* tumor ablation ability. When HIFU is combined with anti-PD-1, tumor proliferation-related genes such as Wnt7b,S100a14 and Erbb2 are significantly downregulated, and newly released tumor-specific antigens, cytokines, and cell fragments act as agonists of innate immunity (84). However, the disadvantage of thermal ablation is that it may enhance the immunosuppressive effect of distant Tregs (84).

Compared with thermal ablation, mild hyperthermia showed a better inhibitory effect on distant metastasis (49, 85). A study by Hongwei et al. reported that PTT combined with anti-CTLA-4 therapy can effectively inhibit the growth and lung metastasis of invasive 4T1 tumors, which could not be achieved by using PTT or anti-CTLA-4 inhibitors alone (86). Interestingly, only one combination of PTT and sequential PTT alone did not cause significant inhibitory effect, or even immune rejection, while the inhibitory effect of sequential PTT combined with anti-CTLA-4 therapy was significant. The reason behind this phenomenon may be explained by a short treatment window after the second dose of PTT treatment. Most of the Treg cells were eliminated and the activated cytotoxic T lymphocytes (CTL) had not infiltrated the tumor tissue. Combined anti-CTLA-4 therapy could accelerate this process and produce stronger TAA-specific CD8+T cells and immune memory than sequential PTT alone. However, when MHT was added to the combination of radiotherapy and anti-PD-1 or anti-CTLA-4, only CD3+T lymphocyte infiltration was observed, which helped reduce the lung metastasis of 4T1 breast cancer but did not improve the overall survival time. Moreover, the combination of MHT and anti-PD-1 or anti-CTLA-4 even showed the possibility of increasing lung metastasis (83). In summary, PTT could be the first to show more potent inhibitory effect than MHT in distant metastasis of breast cancer, suggesting the possibility of a photothermal vaccine (55).

## Pancreatic cancer

Pancreatic cancer is generally considered to be a “cold” malignant tumor of the digestive tract, mainly due to its immunosuppressive “cold” TME (87). At the same time, it is highly metastatic in the early stage because of its incomplete capsule, rich lymphatic and blood circulation, and highly invasive capability (88, 89).

The combination of thermal ablation and mild hyperthermia with ICIs seems to make this “cold” tumor “hot”. PetrosX et al. developed a pulsed high-intensity focused ultrasound (pHIFU) technique based on HIFU. Mechanical damage induced by pHIFU leads to a sustained increase in the level of CD8+ tumor-infiltrating T cells (TILs) in tumors (90). When pHIFU was used in combination with anti-PD-1, the immunosuppression of the PD-1/PD-L1 axis of pancreatic cancer KPC cells was relieved, and the survival rate of the treatment group was significantly increased. In addition, one of the key findings of PetrosX et al., who investigated the combination of thermal ablation and ICIs, was that this combination of therapies induces a local tumor pro-inflammatory microenvironment, which is evidenced by the increased infiltration and proportion of several types of inflammatory cells (CD8+TIL, CD8+ IFN γ + TIL, and CD4+T cells) 48 hours after treatment.

From a cellular structural point of view, the “cold” tumors in pancreatic cancers comprised of dense connective tissue stroma and extracellular matrix, which set up barriers that prevented immune cells from infiltrating to the tumor site (91). Recently, mild hyperthermia has been shown to alter the dense structure of tumor tissue (92). Qianwen et al. designed a PTT therapy based on nanoparticles BMS-202 (a small molecular inhibitor of PD-1/PD-L1). It was found that PTT can dilate tumor vascular morphology and enhance tumor vascular permeability compared to the untreated group, suggesting that this PTT therapy could be beneficial in reshaping the immunosuppressive microenvironment in pancreatic cancer (93). Unsurprisingly, this PTT therapy not only decreased the expression of HIF-1 α and effectively reduced the hypoxia of TME but also promoted the maturation of DC in the spleen of tumor-bearing mice and reactivated the immunosuppressive microenvironment. When combined with nanoparticles BMS-202, the lung and liver metastatic nodules of pancreatic cancer were significantly reduced.

In short, when combined with ICIs, thermal ablation and mild hyperthermia could enhance the therapeutic effect of ICIs and make pancreatic cancer “hot” again by inducing local tumor pro-inflammatory microenvironment and activating tumor immunosuppressive microenvironment, respectively. It is worth mentioning that PTT therapy based on nanoparticles BMS-202 could also inhibit the metastasis of cancer cells through blood vessels to distant organs.

## Nasopharyngeal carcinoma

Nasopharyngeal carcinoma (NPC) is a malignant tumor that often occurs at the top of the nasopharynx. Although it is sensitive to radiotherapy, and simultaneous radiotherapy and chemotherapy are often used in clinics, 30% to 40% of patients die of local recurrence and metastasis, which may be related to radiation resistance (94, 95). At the same time, patients often suffer from many adverse reactions (such as radionecrosis, dysphagia, and vomiting) (96, 97). Hyperthermia can solve the problem of radiation resistance and adverse reactions to some extent, which may be helpful to inhibit the recurrence and metastasis of nasopharyngeal carcinoma.

Recent studies have found that PTT had a good effect in the treatment of nasopharyngeal carcinoma. Qinmin et al. invented a USPIO-PEG-sLex nanoparticle as a photothermal agent for PTT, which proved that it could effectively inhibit the growth and promote apoptosis of nasopharyngeal carcinoma *in vivo* and *in vitro*, and there was no metastasis or invasion in the xenotransplantation model (98). This has also been well established by Naveen et al., and the role of PTT in inducing apoptosis could be related to the surge in ROS level (99). However, after objectively evaluating this method, it might not be possible to use PTT alone for antineoplastic therapy. Hence, to address the question of whether a combination of hyperthermia and other treatments might benefit NPC patients, a retrospective analysis found that the 5-year overall survival rate (OS) of patients treated with whole body hyperthermia (WBH) combined with CRT increased from 65.2% to 80.3% (100) compared with advanced NPC patients who received only radiotherapy and chemotherapy (CRT). More importantly, there was no significant toxicity in these patients, suggesting that WBH helps to improve radiation resistance and reduce therapeutic toxicity in patients with NPC. The effect of the combination of WBH and CRT is gratifying, therefore there are studies on the combination of mild hyperthermia and CRT plus cetuximab to test the efficacy of this triple therapy. The results showed that the apoptosis rate of nasopharyngeal carcinoma CNE induced by triple therapy was further increased on the basis of mild hyperthermia and CRT, and the curative effect was stronger than that of other groups. The mechanism may be related to the synergistic effect of blocking the binding of EGFR and its ligands and the increase of the Bax/Bcl-2 ratio (101). At the same time, this triple therapy has been implemented in pancreatic cancer, and its feasibility has been confirmed, but its safety has yet to be evaluated.

## Brain cancer

Glioblastoma (GBM) is a highly invasive and destructive brain tumor characterized by poor prognosis and high recurrence rate (102). As a result of the tumor microenvironment of restricted blood-brain barrier (BBB) and GBM immunosuppression, immunotherapy, such as ICIs, is ineffective in the treatment of intracranial malignant tumors (103). On the one hand, the lack of TILs and the formation of immunosuppressive mechanism make GBM become a kind of “cold” tumor. On the other hand, monoclonal antibodies are difficult to enter the brain through BBB because of their large molecular size and low BBB permeability.

Laser interstitial thermotherapy (LITT) is a common method for the treatment of recurrent or deep brain tumors. The combination of LITT and ICIs may hopefully change the “cold” state of GBM immunosuppression into a “hot” state that is more sensitive to ICIs. Some studies have generated gold nanoparticles that have a therapeutic action similar to LITT, amplifying the effect of light-based photothermal ablation. When combined with anti-PD-L1, the tumor of GBM model mice reduces in size and the survival rate increases, showing long-lasting anti-tumor immunity (104). At the same time, solving effectively the problem of monoclonal antibody penetrating BBB is the key to improve the efficacy of monoclonal antibody in the treatment of GBM. Recently, MHT has shown good potential, with its mechanism potentially being related to the nanoparticle-encapsulated monoclonal antibodies passing through BBB. The combination of FeNP-based MHT with local injection of nano-adjuvants and systemic injection of anti-CTLA4 can lead to systemic therapeutic responses to inhibit tumor metastasis (75). In addition, nano-drugs, especially NPs combined with hyperthermia, are being studied as potential ways to enhance tumor drug delivery in patients with GBM (105).

In addition to GBM, studies have shown that a common, refractory pediatric cancer called neuroblastoma is sensitive to a combination of Prussian blue nanoparticles (PBNP) photothermotherapy (PTT) and anti-CTLA-4 checkpoint inhibitors. Compared with the group treated with PTT or anti-CTLA-4 checkpoint inhibitor alone, mice treated with this photothermal immunotherapy not only increased the survival rate by 43% or 55.5%, respectively, but also showed protection against neuroblastoma re-attack, suggesting the potential of PTT and ICIs in the treatment of brain tumors (106).

## Thermal ablation or mild hyperthermia combined with ICIs: A clinical study

It has been established that the immune response mediated by T cells released by ICIs is non-specific (107), and thermal ablation may be able to correct this. In a clinical trial to evaluate the efficacy of RFA, patients with liver metastasis from colorectal cancer developed a tumor antigen-specific T cell response after RFA (108). Although RFA does not ablate tumor cells, it offers the possibility of therapy involving a combination of hyperthermia and ICIs. RFA may also make up for the deficiency of nonspecific T cell response induced by ICI (109). Secondly, ICIs therapy often causes adverse reactions. For instance, in patients with advanced melanoma, 96.8% of patients treated with ipilimumab and nivolumab had immune-related adverse events (irAE), and 59% of patients developed grade 3 or 4 irAE. A retrospective study of 131 patients with stage IV melanoma found that when combined with systemic or local hyperthermia, the incidence of grade 3 and grade 4 irAE in these patients decreased to 6.11% and 2.29% respectively, suggesting that mild hyperthermia combined with ICIs is safer (110). This has also been confirmed by a prospective phase I clinical trial. Guoliang et al. found that HT combined with adoptive cell therapy (ACT) and anti-PD-1 therapy was safe and feasible, but this combination of therapies also enriched the TCR library of clinical immune responders and promoted favorable changes in serum IL-2, IL-4, TNF- α and IFN- γ levels (111).

In short, combined with the current evidence, hyperthermia has an advantage in providing a tumor antigen-specific T cell response, while mild hyperthermia combined with ICIs can ensure safety while having anticancer efficacy, and the safety is easy to be ignored (**Table 1**).

**Table 1:** Examples of ongoing clinical studies combining ICIs with hyperthermia therapy.

Trail	Status	Phase	Hyperthermia specifications	Type of neoplasm	Involving metastatic tumors	Treatment	Endpoints
NCT02833233	Active	Pilot	CA	Breast cancer	No	anti-PD-1+anti-CTLA-4+CA	Safety
NCT03237572	Recruiting	I	HIFU, target 50% of the tumor, up to 3 cubic centimeters	Breast cancer	Yes	anti-PD-1+HIFU	Immune response, Safety
NCT04116320	Recruiting	I	Focused ultrasound ablation (FUSA)	Advanced tumor	No	anti-PD-1+HIFU	Immune response, Safety
NCT04156087	Recruiting	II	Minimally Invasive Surgical Microwave Ablation (MIS-MWA)	PC	No	anti-PD-1+MWA+Gemcitabine	PFS
NCT04220944	Recruiting	I	MWA(covered at least two thirds the size of the nodules)	HCC	No	anti-PD-1+MWA/TACE	PFS, ORR, TTP, OS, Safety
NCT03864211	Active	I/II	MWA/RFA under CT or ultrasound guidance	HCC	No	anti-PD-1+MWA/RFA	PFS, OS, Safety
NCT01853618	Completed	I/II	RFA/TACE/CA	HCC	No	anti-CTLA-4+RFA/TACE/CA	Safety, feasibility, RR, TTP, OS
NCT03939975	Completed	II	MWA/RFA	HCC	No	anti-PD-1+MWA/RFA	Safety, RR, PFS, OS
NCT03753659	Recruiting	II	MWA/RFA under CT or ultrasound guidance	HCC	No	anti-PD-1+MWA/RFA	ORR, OS, Safety
NCT04150744	Recruiting	II	RFA	HCC	No	anti-PD-1+RFA	PFS, ORR, OS,TTP
NCT03337841	Unknown	II	RFA	HCC	No	anti-CTLA-4+RFA/Surgery	PFS, OS, ORR, Safety
NCT02821754	Active	II	RFA/CA	HCC、BTC	No	anti-PD-L1+RFA/TACE/CA	PFS, Safety
NCT02469701	Completed	II	CA	NSCLC	Yes	anti-PD-1+CA	RR
NCT02437071	Active	II	RFA	CRC	Yes	anti-PD-1+RFA	Safety, RR
NCT03101475	Completed	II	RFA	CRLM	Yes	anti-PD-L1+RFA+SBRT	Immune response, OS, Safety
NCT03393858	Unknown	I/II	Thermotron RF-8EX, Hyperthermia for 40 minutes on 42°C ± 0.5°C	MM	No	anti-PD-1+MH+DC-CIK	PFS, OS, Safety
NCT03757858	Unknown	I/II	Thermotron RF-8, Hyperthermia for 40 minutes on 42°C ± 0.5°C	Abdominal and pelvic malignant tumor	Yes	anti-PD-1+MH+CAR-T	Safety, ORR, PFS

CA, Cryoablation; HIFU, High intensity focused ultrasound; MWA, Microwave ablation; TACE, Transcatheter arterial chemoembolization; RFA, Radiofrequency ablation; SBRT, Systems Biology Research Tool; MH, Mild hyperthermia; PC, Pancreatic cancer; HCC, Hepatocellular carcinoma; BTC, Biliary Tract Carcinomas; NSCLC, non-small cell lung cancer; CRC, colorectal cancer; CRLM, colorectal cancer liver metastases; MM,Malignant mesothelioma; PFS, Progression-Free-Survival; ORR, Overall Response Rate; TTP, Time to Progression; OS, Overall Survival; RR,response rate. Note: the data in this table is quoted from clinicaltrials.gov.

## Conclusions and future perspectives

Previous studies have mostly focused on the immune effect of hyperthermia, and the combination therapy of hyperthermia and ICIs has been a new field of investigation. This paper first compares the different effects of thermal ablation and mild hyperthermia on immune response, then summarizes the recent examples of preclinical and clinical studies of hyperthermia combined with ICIs, and analyzes the underlying reasons combined with the mechanism of hyperthermia, in order to provide theoretical and experimental basis for research on hyperthermia and ICI combined therapy.

This paper presents some limitations, such as a limited description of the methods and materials used in hyperthermia, whereby there is regular, rapid development of this technique. At the same time, due to the lack of clinical research on the combination of hyperthermia and ICIs, some key questions remain unanswered, such as: how can we better induce anti-tumor immune response with ICIs combined with thermal ablation or mild hyperthermia; at which temperature range does the body have the strongest immunity; what the immune-related factors that affect the efficacy of both are; how safe both techniques are; and how the combined therapy affects the survival rate of patients. These issues need to be addressed in order to greatly improve the outcome of combined therapy with HT and ICIs.

However, combined with the existing evidence, we can still draw some new key conclusions (1): CD8+T cell group is one of the important mechanisms of antitumor immunity in mild hyperthermia, but it may not be as crucial in thermal ablation (2); both thermal ablation and mild hyperthermia can partially improve the immunosuppressive TME and increase the sensitivity of tumor cells to ICIs (112) (3); mild hyperthermia showed a better inhibitory effect on the high metastasis of some tumors, which may be related to the improvement of distant vascular permeability and the promotion of immune cell infiltration (113); and (4) thermal ablationcan provide tumor antigen specific T cell response to ICIs, while mild hyperthermia combined with ICIs can provide safety. However, there are some limitations in thermal ablation, mild hyperthermia or ICIs. The main issue of using thermal ablation or mild hyperthermia itself is the unnecessary heating of healthy tissue and heat tolerance of cells. ICIs has the ability to specifically target cancer cells, which can help some nanoparticles reach the tumor site during hyperthermia, accurately identify and kill cancer cells while protecting normal cells as much as possible. In the clinical treatment of ICIs, treatment-related irAE events often occur, including nausea, diarrhea, loss of appetite, weakness, metabolic abnormalities. Mild hyperthermia reduces the incidence of irAE events, especially for grade 3-4 irAE reaction, which ensures safety while enhances their therapeutic effect. For example, in the clinical application of HIFU, coagulation necrosis caused by excessive ultrasound energy destroys the structure and blood vessels of the tumor to a great extent, thus limiting the ability of immune cells to reach and interact with the tumor. Therefore, some researchers invented p-HIFU technology to reduce ultrasound energy and found that the combination of p-HIFU and anti-PD-1 can promote immune cells to reach the tumor site and induce a local tumor pro-inflammatory microenvironment more effectively. Compared with RFA, MWA and laser, the release of TAA, cytokines and cell fragments caused by HIFU combined with anti-PD-1 acts as an agonist of innate immunity and takes the lead in showing advantages in thermal ablation. At the same time, more research on the combination of CA and ICIs is also needed. The opposite extreme temperature of CA can also cause a stronger immunostimulatory response, which is characterized by a significant increase in the levels of serum IL-1, IL-6, nuclear factor-kappa β and tumor necrosis factor-α after ablation. A preliminary study of patients with breast cancer receiving CA and anti-CTLA-4mAb has shown promising efficacy and good tolerance (114).

Therefore, in the future, hyperthermia and ICIs should be combined as a method to enhance local immune response and avoid systemic immunotoxicity. The goal of the combination of hyperthermia and ICIs will not simply transform the patient’s immunosuppressive state into an activated state but can induce the body’s own immunity. This is beneficial to the low-dose use of ICIs to reduce the occurrence of irAE events, as expected by Edward Jenner (110). In addition, whether hyperthermia and ICIs can make up for each other’s shortcomings and exert synergistic effect also depends on a key question-whether a TME characterized by PD-L1 overexpression and TIL enrichment can be produced (60). The enrichment of TILs by hyperthermia has been confirmed (115, 116), but there are limited studies on the expression of PD-L1 or other immune checkpoints during hyperthermia. If hyperthermia can upregulate the expression of PD-1, PD-L1 or CTLA-4, it could be used as an ICIs sensitizer to bring improved efficacy to clinical malignant tumor patients while explaining the mechanism behind it, which is very important.

## Author contributions

XY performed the manuscript preparation and drafted the manuscript. MG, RX, YT helped draft the manuscript. WL, WZ, LH, BW revised the manuscript and approved the final version. YCH contributed to the conception and design of the current study, revised the manuscript, and approved the final version. All authors contributed to the article and approved the submitted version.

## Funding

This work was supported by the National Natural Science Foundation of China (grant nos. 81874408), the Project of the Hunan Provincial Department of Education (grant nos. 21B0359), the Project of Hunan Provincial Health Commission (grant no. 202207015643), and the Domestic First-Class Discipline Construction Project of Chinese Medicine of Hunan University of Chinese Medicine (grant nos. 2022ZYX03).

## Acknowledgments

We would like to thank Editage (www.editage.cn) for English language editing.

## Conflict of interest

The authors declare that the research was conducted in the absence of any commercial or financial relationships that could be construed as a potential conflict of interest.

## Publisher’s note

All claims expressed in this article are solely those of the authors and do not necessarily represent those of their affiliated organizations, or those of the publisher, the editors and the reviewers. Any product that may be evaluated in this article, or claim that may be made by its manufacturer, is not guaranteed or endorsed by the publisher.
